# The interrelationship between cerebral ischemic stroke and glioma: a comprehensive study of recent reports

**DOI:** 10.1038/s41392-019-0075-4

**Published:** 2019-10-12

**Authors:** Mrinal K. Ghosh, Dipankar Chakraborty, Sibani Sarkar, Arijit Bhowmik, Malini Basu

**Affiliations:** 1 Signal Transduction in Cancer and Stem Cells Laboratory, Division of Cancer Biology and Inflammatory Disorder, Council of Scientific and Industrial Research-Indian Institute of Chemical Biology (CSIR-IICB), 4 Raja S.C. Mullick Road, Kolkata 700032 and CN-06, Sector-V, Salt Lake, Kolkata, 700091 India; 2 Department of Cancer Chemoprevention, Chittaranjan National Cancer Institute, 37 S. P. Mukherjee Road, Kolkata, 700 026 India; 3 Department of Microbiology, Dhruba Chand Halder College, Dakshin Barasat, South 24, Paraganas, 743372 India

**Keywords:** CNS cancer, Cancer microenvironment

## Abstract

Glioma and cerebral ischemic stroke are two major events that lead to patient death worldwide. Although these conditions have different physiological incidences, ~10% of ischemic stroke patients develop cerebral cancer, especially glioma, in the postischemic stages. Additionally, the high proliferation, venous thrombosis and hypercoagulability of the glioma mass increase the significant risk of thromboembolism, including ischemic stroke. Surprisingly, these events share several common pathways, viz. hypoxia, cerebral inflammation, angiogenesis, etc., but the proper mechanism behind this co-occurrence has yet to be discovered. The hypercoagulability and presence of the D-dimer level in stroke are different in cancer patients than in the noncancerous population. Other factors such as atherosclerosis and coagulopathy involved in the pathogenesis of stroke are partially responsible for cancer, and the reverse is also partially true. Based on clinical and neurosurgical experience, the neuronal structures and functions in the brain and spine are observed to change after a progressive attack of ischemia that leads to hypoxia and atrophy. The major population of cancer cells cannot survive in an adverse ischemic environment that excludes cancer stem cells (CSCs). Cancer cells in stroke patients have already metastasized, but early-stage cancer patients also suffer stroke for multiple reasons. Therefore, stroke is an early manifestation of cancer. Stroke and cancer share many factors that result in an increased risk of stroke in cancer patients, and vice-versa. The intricate mechanisms for stroke with and without cancer are different. This review summarizes the current clinical reports, pathophysiology, probable causes of co-occurrence, prognoses, and treatment possibilities.

## Graphical abstract

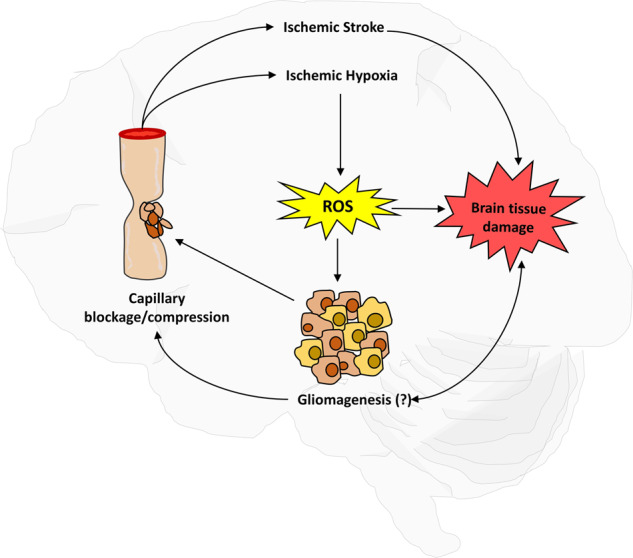

## Introduction

Cerebral ischemia, also known as cerebral ischemic stroke or cerebrovascular ischemia, is the most common type of stroke (>80%) and is the second leading cause of death, dementia, and disability worldwide.^1^ This condition occurs when a sudden obstruction of the blood supply or a reduction of normal cerebral blood flow (CBF) leads to brain injuries.^2^ The sudden brain tissue damage due to a low supply of nutrients and hypoxia is also known as cerebral infarction and is further divided into two major categories according to origin. Focal cerebral ischemia, micro-ischemia, or local cerebral infarction is caused by blockage of a blood vessel^3^ due to onsite blood clot formation (thrombus)^4^ or a blood clot that originates in a different place (embolus, sporadic),^5^ whereas the global cerebral ischemic condition originates due to hypoperfusion or a drastic reduction of CBF in the overall brain caused by large-artery atherosclerosis, complete obstruction of the carotid arteries, cardiac arrest, chronic hypoxemia, or seizures.^6^

Several intracranial malignant tumors occur in the human brain, of which glioma is the deadliest and rarely curable form and is resistant to radiotherapy and chemotherapy^7^ According to the World Health Organization (WHO), glioma can be classified into four different grades (I–IV), where grade I includes pilocytic astrocytoma, grades II–III include diffuse or anaplastic astrocytoma and oligodendrogliomas, and grade IV includes most malignant glioblastomas (GBMs).^8,9^ Despite the major driver mutations (TP53, IDH1, EGFR, PTEN, Rb, RTKs, and others), several mechanical or molecular signaling alterations are found in all grades of glioma and within its microenvironment.^10^ The postoperative approximate survival time for GBM patients is ≤15 months, and only 26.5% of patients survive for >2 years after diagnosis.^11^

In this review, our major aim is to document the interrelationship between cerebral ischemic stroke and glioma based on a comprehensive review of current knowledge, which is sequentially discussed in detail. First, we briefly discuss the relationship between cerebral ischemia and glioma that could explain the interplay between the two diseases. Second, we discuss the effects of cerebral ischemia on glioma development and progression. Third, we elaborate on the effects of reactive oxygen species (ROS), reactive nitrogen species (RNS), and the neurovascular unit on brain tumors. Fourth, we focus on glioma-dependent cerebral ischemic stroke and brain injuries. Finally, plausible pharmacological interventions towards therapeutic strategies are discussed.

## Interplay between cerebral ischemia and glioma: what do clinical reports reveal?

The relationship between cerebral ischemia and glioma is still ambiguous based on molecular mechanisms, but several clinical reports and case studies have indicated that glioma and cerebral ischemia can facilitate each other with respect to occurrence. It has been reported that the location of the tumor inside the brain (insula, operculum, and temporal lobe) and repeated resection during glioma therapy can increase the risk of ischemic injuries and other neurological deficits.^12^ A recent report based on clinical cohort studies suggests that the chance of the diseases occurring together reaches 9% compared with 2.7% in the control population, and the risk of developing brain cancer (especially glioma) is also higher in stroke patients.^13^ Another clinical cohort-based study on 3680 noncancerous adults with no disabling cerebral infarction reported the development of brain cancer (glioblastoma) with a mortality rate that is threefold higher than that of the control cohort in the postischemic period.^14^ Another case study of a 73-year-old woman with a history of atrial fibrillation and mechanical aortic valve replacement showed primary glioma development within the territory of a previous ischemic infarction.^15^ A similar result of the sudden onset of an acute ischemic lesion near the tumor area was reported in another case study of a 77-year-old woman suffering from an anterior temporal lobe tumor.^16^ A different report stated that two adult patients with supratentorial glioblastomas developed an ischemic stroke on the tumor site.^17,18^ A recent case study reported that the risk of neurodegeneration and ischemic lesions increases after resection of recurrent tumors.^19^ The case of an anaplastic astrocytoma patient showed acute onset ischemic stroke-like symptoms.^20^ In another interesting case, a 79-year-old woman with a history of atrial fibrillation and coronary heart disease developed glioblastoma multiforme (GBM) at the site of a previous infarction 6 years after the onset of right hemiplegia.^21^ Cerebral ischemia might occur due to embolus metastatic glioma cells, as reported recently.^22^ Another unusual case of acute ischemic infarction of the middle cerebral artery was caused by a proliferating glioma mass.^23^ In certain cases, it is notably difficult to distinguish the early symptoms of stroke and glioma, which might lead to improper therapy. Several reports worldwide present these pseudo-symptoms of glioma and cerebral ischemia.^24,25^ Another interesting case is a woman from India who was primarily diagnosed as a cerebral stroke patient but was later found to exhibit glioma development instead of stroke symptoms.^26^

**Fig. 1:**
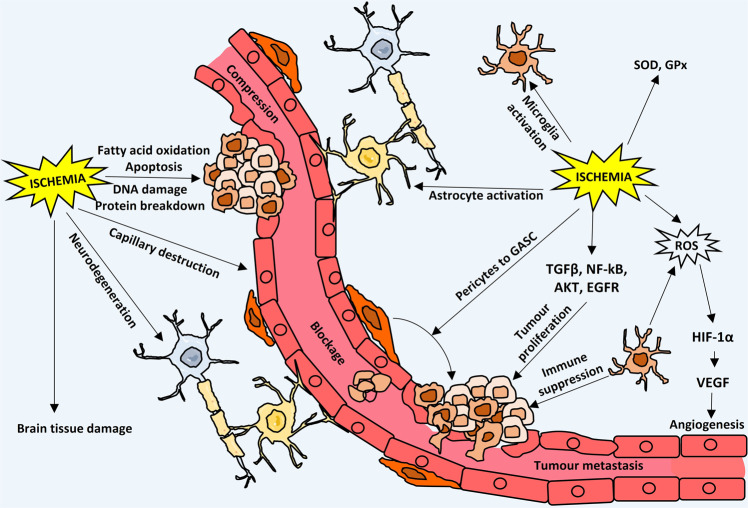
Bi-phasic role (i.e., detrimental and tumor promoting) of cerebral ischemic hypoxia and glioma development

The most widely accepted model that connects ischemia and glioma is based on the common hypoxic condition that occurs in both situations.^13,19,27,28^ Cerebral ischemia due to obstruction in the vasculature locally or globally causes low oxygen tension in the ischemic regions and results in hypoxia, whereas a highly proliferating glioma cell mass has poor vasculature inside its core, leading to a hypoxic core region that is deprived of oxygen.^29^ The exact mechanisms of this co-occurrence or interplay are still in the nebulous phase, but certain possible mechanisms, e.g., astrocyte activation,^30,31^ reactive gliosis,^32–34^ angiogenesis^35–37^, and changes in perivascular and perinecrotic niches^38–40^ due to cerebral ischemia, are reported as a consequence for glioma development. In this review, all of the possible methods of interplay are described in a sequential manner (Fig. 1).

## Cerebral ischemia, hypoxia and glioma: ROS and RNS connection

ROS are metabolic byproducts, e.g., hydroxyl radicals (HO•), alkoxyl radicals (RO•), hydrogen peroxide (H_2_O_2_), and hydroperoxyl radicals (HO_2_•), originating from different sources in hypoxic^41^ and hyperoxic situations with condition-dependent functions.^42–44^ Multiple sources for ROS production are reported in both ischemia and glioma, and both conditions share a common network of signaling for ROS production and downstream functions.

In the cerebrovascular unit, the hypoxic situation induces astrocytes, microglia, pericytes, and even neurons to produce ROS and RNS (NO, ONOO^−^).^45–47^ ROS, together with RNS, take a lead role in regulation of the hypoxic situation in the affected tissue mass.

### Mitochondrial ECT

In mitochondria, electrons flow into the sequential Electron transport chain (ETC) (complex I, II, III) and ultimately meet up with O_2_ at complex IV for ATP synthesis and H_2_O production via the oxidative phosphorylation (OXPHOS) process.^48,49^ However, in the case of oxidative stress or other pathophysiological conditions, more leaky electrons are produced, and ROS are mainly generated from complex I, III, and glycerol 3-phosphate dehydrogenase.^50^ During oncogenesis, several cancer-specific external stimuli or signaling alterations (e.g., TNF-α, STAT3) cause a decrease in the mitochondrial membrane potential that hampers the components of ETC such that ROS generation is promoted on a large scale.^51,52^ Reports exist of mutation in components of the ETC complexes, and mtDNA can cause a high level of ROS production.^53,54^

### Cytoplasmic NOX

The NADPH oxidase (NOX) family of proteins is one of the main producers of ROS in several cancers and ischemic stroke.^55^ NOXs are membrane-bound proteins with a C-terminal NADPH/FAD-binding domain and N-terminal transmembrane tandem heme groups.^56^ It is reported that NOX2 (gp91phox) and its homologs (Nox1, 3, 4, and 5 and Duox1/2) require p22 phox as a cosubunit and catalyze superoxide (O_2_^−^) generation via a NADPH-dependent pathway, which is subsequently converted to H_2_O_2_.^57,58^ However, Duox1/2 has an EF-hand domain for calcium binding instead of the heme group.^59^ Specific signals (viz., TGF-β,^60^ AKT,^61^ PKC,^62^ MAPK, ERK, etc.) induce conformational changes in the NOX complex in a phosphorylation-dependent manner and allow production of a notably large amount of ROS.^63^

### Peroxisomes

The peroxisome is one of the major sites for α- and β-oxidation of fatty acids, polyamine oxidation, phospholipid and glyoxalate metabolism, catabolism of amino acids, the pentose phosphate pathway, etc.^64^ The peroxisome contains several enzymes (e.g., Acyl-CoA oxidases, d-amino acid oxidase, urate oxidase, aspartate oxidase, polyamine oxidase, xanthine oxidase (XO), l-alpha-hydroxy acid oxidase, pipecolic acid oxidase, trihydroxycoprostanoyl-CoA oxidase, etc.) that produce H_2_O_2_, O_2_^−^, and OH• as normal metabolic byproducts.^65^ The antioxidant defense system inside the peroxisome maintains homeostasis against those ROS, but in ischemia and glioma, this homeostasis is disturbed due to oxidative stress, which alters signaling and mutation and produces overactivation of several enzymes inside this organelle to give rise to an increased level of ROS inside the cell.^66,67^

### Xanthine oxidase

XO is a homodimeric metalloprotein with one flavin adenine dinucleotide (FAD) cofactor for purine oxidation and a molybdopterin cofactor (Moco) for NAD^+^ reduction flanked by two nonidentical iron-sulfur redox centers.^68,69^ In glioma and ischemia, the hypoxic condition and low pH allow XO to form a large amount of H_2_O_2_, O_2_^−^, and OH• via the Haber–Weiss–Fenton reaction.^70–72^

### Cytochrome P450 (CYP)

CYPis a monooxygenase with a heme (FeIII) prosthetic group,^73,74^ and its isoforms in different regions of the body regulate the biotransformation pathway of several endogenous and exogenous toxins, chemicals, xenobiotics, and organic molecules. This system can generate different ROS species (H_2_O_2_, O_2_−, •O_2_^−^, OH^−^) via abnormal uncoupling of the normal metabolic pathways due to hypoxia-specific signals.^75–77^

### Lysyl oxidases (LOXs)

Protein-lysine 6-oxidase, also known as LOX, produces H_2_O_2_ as a byproduct during crosslinking between cell-matrix protein elastin and collagen using the lysyl tyrosylquinone cofactor.^78^ The enzyme is regulated by Hif-1 or Hif-2 and generates ROS and induces metastasis and cell-matrix adhesion via the FAK/Src signaling pathway in both ischemia and glioma.^79–82^

### Involvement of other signaling pathways in ROS and RNS generation

**Fig. 2:**
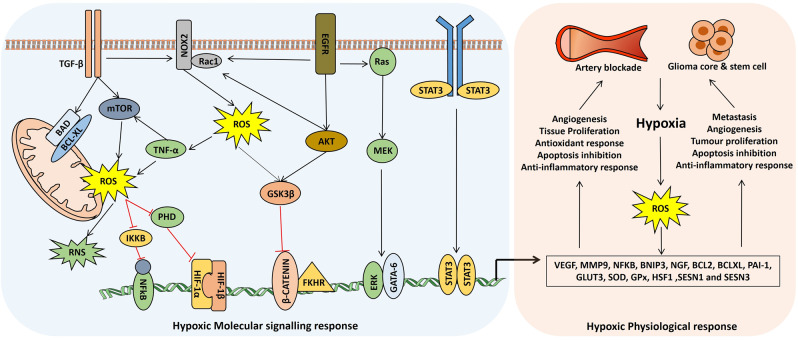
Common hypoxic signaling pathways for cerebral ischemia and glioma

ROS can be regulated by the Ras–Raf–MEK pathway via transcriptional regulation of Nox1 by the GATA-6.^83,84^ It is also reported that transcriptional enhancement of HSF1 by Ras upregulates the *SESN1* and *SESN3* genes and peroxiredoxins for ROS production.^85^ TGFβ increases ROS production via activation of GSK3β and the mTOR pathway in mitochondria, and by suppressing antioxidant enzymes such as SOD and glutathione peroxidase (GPx).^86,87^ Nuclear factor-κB (NF-κB) can increase ROS production via a positive feedback loop of TNF regulation.^88,89^ c-Myc can regulate ROS production via two mechanisms, i.e., ROS production via alteration of mitochondrial structure and metabolism with the aid of AMPK and PRx-Romo1 pathway regulation.^90–92^ It is also reported that the ROS level can be upregulated by the β-adaptin/c-Myc pathway.^93^ The PI3K/mTOR and STAT5 pathway is activated by Bcr-Abl to increase mitochondrial ROS production^94,95^ (Fig. 2).

However, ischemic hypoxia-induced constitutive or inducible nitric oxide (NO) production is enhanced due to glutamatergic receptor-mediated high calcium concentration and calmodulin-dependent upregulation of nitric oxide synthase (nNOS, eNOS, and iNOS).^96,97^ Peroxynitrite (ONOO^−^) is generated by the reaction of NO and ROS.^98^ Neuronal NOS (nNOS) is constitutively active and produces a low amount of NO from neurons, but it kills the surrounding non-NOS-containing neurons. NO produced by endothelial NOS (eNOS) is a vasodilator and has neuroprotective properties. NO from the induced NOS (iNOS) is the main culprit for cerebral ischemic damage and kills the endothelium by 3-nitrotyrosine formation under oxygen and glucose deprivation.^99,100^

## Effect of ischemic ROS and RNS on Glioma

Surprisingly, ROS and RNS have a dual role in the neurovascular unit, where they destroy tissues and macromolecules during the detrimental phase (global ischemia, reperfusion injuries) and aid in cell proliferation, tissue repair and regeneration and angiogenesis in the recovery phase (acute ischemic stroke, hypoxic tumor core, perivascular niche (PVN)).^101^ Moderately increased ROS are oncogenic while the highly increased level of ROS acts as a tumor suppressor.^102^ Therefore, cells bearing high levels of ROS are more susceptible to death, and the opposite is also true for the depleted ROS level in tumors. Furthermore, the level of cellular ROS is increased due to depletion of antioxidants and potentially contributes to the oxidative damage to biological macromolecules that leads to cytotoxic and mutagenic responses. ROS can contribute to genomic instability, thereby resulting in cell death or tumorigenesis. At the threshold level, ROS are recognized as intracellular signal transduction molecules that regulate kinase-driven pathways and mediate cellular responses to external stimuli. Additionally, ROS inhibit many phosphatases that negatively regulate signaling cascades, whereas an increased level of cellular ROS during oxidative stress creates an oxidant/antioxidant imbalance and is responsible for several malignancies. Based on the amount and potential, hypoxic ROS either aid in tumorigenesis and recurrence or cause massive tissue damage.

### ROS favor tumor growth

The ROS-induced signaling pathways, viz. EGFR, MAP kinase,^103,104^ TGFβ,^60,105^ and NF-kB,^106,107^ aid tumor development and progression as also participate in tissue repair, regeneration, and the healing processes in the postischemic recovery phase. ROS can also activate ERK1/2 signaling in glioma.^108^ RAS, an upstream activator of the ERK1/2 pathway, is also activated through oxidative modification by ROS at its cysteine 118 residue, which leads to inhibition of GTP/GDP exchange.^109^ Moreover, ROS can modulate pro-apoptotic factors such as Bax,^110^ Bad, Bim, and FOXO family transcription factors.^111^ Tumor necrosis factor (TNF) and neuronal growth factor (NGF) display various functions from cell growth and differentiation to cell death. TNF-induced ROS can also activate antiapoptotic pathways due to activation of the transcription factor NF-κB. TGFβ is one of the major signaling pathways in both glioma and ischemia.^112,113^ In hypoxic ischemia, the elevated expression of cytokine-mediated TGFβ^114^ upregulates antiapoptotic Bcl2 and Bcl-xl^115^ proteins and PAI-1 (ref. ^116^) and also transactivates the MAPK pathway and offers neuroprotection from oxidative ischemic injuries.^117^ Moreover, in glioma, TGFβ increases ROS production and activates GSK3β via the mTOR pathway in mitochondria by suppressing antioxidant enzymes such as SOD and GPx.^118^

Another important pathway that acts on glioma and hypoxic ischemia in a similar manner is hypoxia-inducing factor 1 (HIF-1). HIF-1 is a heterodimeric protein with two subunits, viz. HIF-1α and HIF-1β, that sense low oxygen tension in the tissue microenvironment and are upregulated due to the inhibition of degradation via PHD inactivation.^119,120^ HIF-1α upregulates the expression of glucose transporter 3 (GLUT3),^121^ VEGF,^122^ erythropoietin,^123^ and BNIP3 (ref. ^124^) and suppresses cytochrome *c* release, PARP cleavage,^125,126^ and p53 activation.^127^ Therefore, in one way, HIF-1 confers cell survival and in other way, it drives angiogenesis.

### ROS are detrimental for tumor growth

Mitochondria are ROS generators that also increase the level of ROS which causes mitochondrial dysfunction.^128,129^ ROS also have great detrimental effects. A high level of ROS promotes severe cellular damage and even cell death. ROS are derived from endogenous and exogenous sources in ischemic stroke as a result of oxidative stress after a stroke, which leads to lipid peroxidation, DNA damage, protein degradation, and apoptosis. Apoptosis can be induced by both intracellular and extracellular signals through two major pathways, namely, the mitochondrial (intrinsic) and death receptor-mediated (extrinsic) pathways.^130^ The intrinsic apoptotic cascade associated with changes in the permeability of the outer mitochondrial membrane and ROS directly trigger this pathway by interacting with the pathway molecules.^131^ ROS induce pro-apoptotic molecules such as p53 and p38 kinases and increase cellular apoptosis.^132^ The truncated form of Bid proteins causes Bax/Bak oligomerization and creates megapores in mitochondria through the direct involvement of ROS, and an apoptosome complex is subsequently formed in the cytosol by activating caspase 9 and 3 to initiate apoptosis.^133^ Apoptosome complex is regulated and influenced by ROS in various contexts. In oxidative stress, excessive ROS are produced that damage biological macromolecules, viz. proteins, lipids, and DNA, creating fatal conditions in tissue cells that contribute to many diseases, including cancer. Increased expression of the Fas receptor or triggering of the mitochondrial permeability transition with the release of ROS is the basic mechanism of apoptosis induction in tumor cells.^134,135^ Intracellular ROS accumulation obstructs cellular proliferation and induces cell cycle arrest at the G1 and G2/M phases.^136,137^ Abnormally increased levels of ischemic ROS can selectively kill malignant cells and act as an adverse factor in causing genetic instability. Thus, enhanced ROS production in the tumor bed might be one of the important strategies in ROS-mediated cancer therapy.

## Role of neurovascular unit in ischemia and glioma

### Astrocytes

Astrocytes are the star-shaped and most abundant housekeeping non-neuronal cells found in the brain microenvironment. These cells form the blood–brain barrier (BBB) and tripartite synapses, help neurons and glial cells by supplying nutrition and other factors from the vasculature, and also maintain communication between the cells and the microenvironment.^138^

In cerebral ischemia, due to oxygen-glucose deprivation, dramatic changes (such as swelling, cytoplasmic hypertrophy, accumulation of GFAP, Vimentin, and other intermediate glial filaments) occur in astrocytes and increases of cellular organelles like mitochondria, ribosomes, nuclear size, and Golgi complexes leads to a metabolically activated reactive form.^139^ A meshwork of the cytoplasmic processes of activated astroglial cells form a glial scar around the area of the ischemic lesions.^32,140^ This activation process and subsequent mutational events of several genes, such as neurofibromatosis type 1 (NF1)^141–143^ and glycoprotein podoplanin (PDPN),^144^ in reactive gliosis lead to gliomagenesis because both glial progenitor and reactive astrocyte cells are proposed origins of the same lineages.^145–147^ Astrocytic STAT3 increases MMP2 expression and inhibits RhoA and PTEN via miR-21, which leads to adhesion turnover, actomyosin tonus, and migration of reactive astrocytes to form a glial scar.^148,149^ These reactive astrocytes enhance uncontrolled proliferation and migration of glioma cells by expressing MMPs^150^ and secretory SDF1.^151–153^ The direct interaction between reactive astrocytes and glioma cells by tunneling nanotubes (TNT) and the secretion of IL6, IL19, IGF1, TGFβ, MCP4, VEGF, etc. aid glioma cells in infiltrating the surrounding parenchyma.^154–156^ The expression of connexin 43 (Cx43)^157^ and different ATP-dependent ion channels (ClC-3, VGCC, TRPs, hERG, ENaC, CLICs)^158^ in reactive astrocytes offer protection against radio- and chemotherapy via activation of Bcl2 family proteins and inhibition of cytochrome *c* release from mitochondria.^159^ It is also reported that reactive astrocytes supply a suitable microenvironment for the transformation of CD133^+^ glioma stem cells from CD133^−^ cells.^153^ Interestingly, the astrocyte’s glycogen stores and the presence of a high amount of metallothionein (MT), glutathione, and other antioxidants protect the surrounding tissues from hypoglycemic and hypoxic ischemic shock.^160–162^

### Microglia

Microglia (CD45^low^, CD68^+^, Iba1^+^) are mononuclear resident phagocytic macrophages of normal brain originated from myeloid stem cells in the yolk sac.^163,164^ The main functions of microglia are to offer immune protection of the brain, a clean brain microenvironment via phagocytosis of unwanted debris, support of other glial cells and neurons, and aid in BBB and synaptic plasticity maintenance.^165,166^ Microglia are one of the major sources of ROS, pro-, and anti-inflammatory cytokines, neurotropic and growth factors and act as the first line barrier of innate immunity by expressing pattern recognition receptors (TLRs, NLRs, and RLRs) for pathogen-associated molecular patterns and danger-associated molecular patterns (DAMPs) recognition.^167–169^ Microglia are involved in several cell signaling networks, e.g., NF-κB, TNFα, TGFβ, interleukin signaling (IL1β, IL6, IL4, and IL10), chemokine receptor signaling (CX3CL1/CX3CR1 and CCL2/CCR2), neurotransmitter signaling, and most importantly TREM2 signaling.^170–173^

Upon ischemic injuries, resident microglia together with monocyte-derived microglia [infiltrating from circulation to the brain tissue via ruptured BBB] become activated to various reactive forms. Interestingly, different classes of these reactive forms act in opposite manners to each other according to the situation. Due to breakdown of glia–neuron communication (CX3CL1/CX3CR1) and several excitotoxic signals such as DAMPs, purinergic signals and acute inflammatory environment resident microglia are transformed into three distinct morphological types, viz. enlarged cell body with low ramifications, amoeboid structure with rare ramifications, and a round-shaped highly activated form distributed from the peri-infarct regions into the core ischemic lesions.^168,174–176^ Despite the morphology, microglia are polarized into two distinct functional phenotypic variants, i.e., pro-inflammatory M1 and anti-inflammatory M2 forms (further divided into M2a capable of repair, immunoregulatory M2b, and immunomodulatory M2c). The classical M1 (CD16^+^, CD86^+^, FcγR^+^, iNOS^+^) phenotype secretes excessive amounts of ROS, RNS, TNFα, IL6, and IL1β for inflammatory response, cytotoxicity, and brain tissue damage. Alternatively, M2 (Arg1^+^, CD36^+^, CD206^+^, Ym1^+^) phenotypes, mostly found in the ischemic core region, secrete IL4, IL10, and IL13 and TGFβ, IGF1, NGF, and BDNF for neuroprotection, inhibition of apoptosis and necrosis, tissue and ECM repair and cleanup of debris via phagocytosis.^177–180^ Transformation of these microglia from M1 (tumor suppressive) to M2 (tumor promoting) form initiates immune suppression in the tumor area and also promotes tumor expansion, metastasis, angiogenesis, and glioma stem cell maintenance via the secretion of several factors (viz. MMPs, CCL18, CCL22, CXCL12, IL10, TGFB, TNF, FasL, VEGF).^181–185^

### Pericytes

Pericytes (PDGFRβ^+^, CD13^+^, NG2^+^, *α*-SMA^+^, Desmin^+^) or Rouget cells are contractile cells located directly on small blood vessels, including capillaries, pre-capillary arterioles, and postcapillary venules.^186^ The major functions of pericytes are formation of blood vessels, glial scars, and the BBB, capillary diameter, and cerebral blood flow (CBF) regulation, amyloid β clearance, and neuroinflammation suppression, and they at times exhibit stem cell-like properties.^187^

In acute focal cerebral ischemia, the “no-reflow phenomenon” and secondary hypoperfusion occur due to structural changes of the ischemic capillary bed because of astrocytic endfeet and endothelial swelling and constrictions of the capillary pericytes.^188,189^ Several pathways, especially ROS-mediated translocation of myosin, thromboxane A2 release, and cytosolic calcium increase, cause pericytes constriction and death after ischemic stroke.^190^ However, ischemic hypoxia results in activation of A2a receptors, and the NO/guanylate cyclase pathway leads to the dilation of pericytes.^191^ Interestingly, pericytic ICAM-1 guides leukocyte migration through gaps between adjacent pericytes during ischemia.^191,192^ Due to induction of TNF-α in the ischemic region, RGS5-expressing pericytes take on an amoeboid morphology, detach from the basal lamina, and migrate toward the ischemic lesion via secretion of MMP9.^193–195^ It is also reported that the phagocytic behavior of pericytes increases during ischemic insults. Pericytes express a variety of neurotropic and neuroprotective factors such as GDNF, BDNF, NGF, and NT-3 that facilitate neuronal and axonal regeneration.^196,197^ Pericytes express Ang1 and GDNF, which maintain and enhance the tight junctions of endothelial cells by up-regulating claudin-5.^198,199^ Pericytes increase angiogenesis via the interactions of VEGF and FLT1,^200^ Ang1 and Tie2,^201^ and PDGFR-β and TGF-β1.^202,203^ Several reports exist on the reprogramming of pericytes into neurons (NG2, sox2, and ascl1)^204,205^ and other glial (Iba1^+^, Glast^+^) cells and formation of a glial scar due to the induction of a lineage-specific stem cell marker in ischemic conditions.^206,207^ These active pericytes aid in immune suppression, remodeling of PVN, and protection of glioma stem cells (GSC) or glioma-initiating cells (GICs) from ischemic injuries.^208,209^ Additionally, GSC recruits vascular pericytes via SDF1/CXCR4 signaling for angiogenesis.^210,211^ GICs maintain self-renewal and differential properties by interacting with pericyte-derived endothelial cells via PDGF-NOS2-ID4 signaling.^212,213^

### Glioma stem cells

GSCs and glioma-associated stem cells (GASC) are two types of cancer stem cells (CSCs) found in the glioma microenvironment.^214,215^ Both cell populations have enhanced self-renewal and differential proliferation properties, but only GSCs can initiate tumor formation and proliferation. GSCs are heterogeneous in origin, are found in the inner core of the tumor mass, express several markers (SOX2, NANOG, BMI1, OLIG2, MUSASHI1, and CD133), and are resistant against chemo- and radiotherapy.^216,217^ These cells interact with the surrounding microenvironment, regulating multiple signaling networks such as VEGF, NF-kB, EGFR, HIF1α, TGFβ, BMP, and NOTCH for promotion of tumor growth, metastasis and angiogenesis.^218^ In contrast, GASC are nontumorigenic tumor supporting stem cells originated from mesenchymal stem cells mostly found in the perivascular area. GASCs are classified into two categories according to their marker profile and functions. A high rate of proliferation of CD90^high^ GASC and secretion of exosomes loaded with growth factors, IL10, miRNA, CCL5, SDF-1α, and MMP9 support glioma proliferation and infiltration, whereas CD90^low^ GASC produces VEGF, IL6, and FGF and is transformed into CD31^+^ from CD13^−^ pericytes for angiogenesis.^219–221^ A major hallmark of glioblastoma is the presence of ischemic pseudo-palisading necrosis, where chromodomain helicase DNA‐binding protein 7 (CHD7) is expressed in an ischemic hypoxia-dependent manner and regulates angiogenesis.^35^ GSCs produce NO via overexpression of nitric oxide synthase-2 (NOS2) in an ischemic condition, which aids in hyperproliferation.^222^

### Blood–brain barrier

BBB is a highly selective physical barrier that regulates direct and indirect diffusion of molecules from circulation into the brain. The BBB consists of a nonfenestrated endothelial cell monolayer of blood capillaries connected by tight junctions and a basement membrane composed of specialized ECM, astrocyte endfeet, pericytes, neurons, and microglia.^223,224^ In hypoxic acute ischemia or high-grade glioma, a high rate of metabolism requires a high oxygen and nutrient supply such that expression of VEGF and PDGF increases, leading to angiogenesis.^225,226^ Increased vascularization together with the altered BBB forms the blood–brain tumor barrier (BBTB) or blood tumor barrier (BTB) with three distinct types of blood capillaries, viz. nonfenestrated continuous normal brain capillaries, continuous and partially fenestrated capillaries, and capillaries composed of inter-endothelial gaps and fenestration.^227^ Altered aquaporin expression and displacement of astrocyte endfeet,^228^ depletion of normal pericytes and recruitment of GSC derived pericytes,^229^ bradykinin-dependent migration of glioma cells toward capillaries,^230^ and finally, degradation of tight junction proteins of endothelial cells alter the BBTB structure and make it leaky, which causes rapid metastasis.^231^ Interestingly, the transmembrane proteins, e.g., ABC transporter, HB-EGF, PTGS2, ST6GALNAC5, and other drug efflux transporters, are also found in the BBTB, which supports chemo-resistance.^232–235^ Another important component of the glioma microenvironment is the PVN at the border area of the tumor and vasculature and is enriched with GSCs.^236^ Several noncancerous cells such as macrophage, pericytes, astrocytes, and endothelial cells give support to GSCs for maintenance and proliferation in this region and maintain an immunosuppressive hypoxic environment. Signaling crosstalk between these cells in PVN makes this region radiotherapy- and chemotherapy resistant.^237–239^

## Glioma leads to ischemic stroke and brain injuries

In glioma, the highly proliferating cell mass, metastasis, BBB breakdown and release of micro- and macroparticles in circulation cause thrombosis and capillary blockade, resulting in the focal ischemic condition.^240,241^ Blood vessel compression due to brain tumor formation also results in cerebral ischemia, which leads to a limited supply of nutrients to the brain that is unable to meet the metabolic demands of the brain tissue. Tumors in the brain progress gradually with time, whereas stroke occurs due to a certain blockage of blood in the brain.^242^ A recent patient cohort-based study on extracellular vesicles shows high correlation with D-dimer levels and cancer, which indicates increased risk of stroke in cancer patients.^243,244^ It is well established that glioma cells release factor X, mucins,^245^ podoplanin,^246,247^ and other procoagulant factors and cytokines^248^ that activate monocytes, endothelial cells, and platelets and also stimulate neutrophils to form neutrophil extracellular traps and inhibit protein C activation, leading to local inflammation and ischemic hypoxia.^249^ Several reports showed that glioma therapy, especially platinum-based drugs, angiogenesis inhibitors, monoclonal antibodies, and radiotherapy, increased the risk of thromboembolism.

**Fig. 3:**
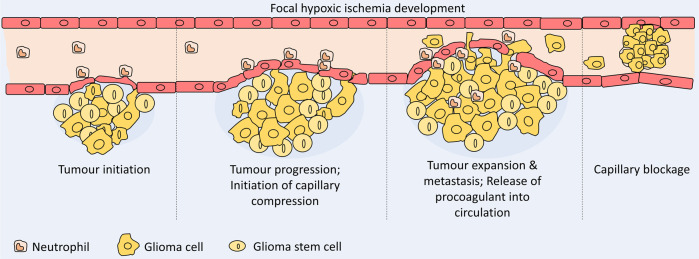
Steps of focal cerebral ischemia development

The characteristics of cancer-related stroke are completely different from those of conventional stroke. Hemorrhagic stroke can cause direct adverse effects on the tumor within the cranial vault.^250,251^ The intravascular coagulopathy that causes embolism is the main mechanism of cancer-related stroke.^252,253^ Direct effects either from tumor compression or from tumor embolism are another causal mechanism of stroke. Tumor bed edema leads to ischemia or infarction in the territory of the affected vessels and is clinically different from tumor progression.^254^ This mechanism is unique in that radiation treatment on the brain tumor might result in a stroke in certain cases. Selected chemotherapeutic agents (viz. cisplatin, methotrexate, l-asparaginase)^255,256^ and antiangiogenic agents (viz. paclitaxel, angiostatin)^257–259^ have also been associated with cerebral stroke. For example, the treatment of GBM with Bevacizumab shows a stroke rate of 1.9%.^260–262^ (Fig. 3).

## Therapeutic approaches for ischemia and glioma

After the onset of cerebral ischemia, oxidative stress plays a major role in neuro-inflammatory diseases.^263,264^ In the postischemic brain, free radicals are increased by redox reactions and express several pro-inflammatory genes by multiple transcription factors, such as NF-κB, and inhibit the cellular antioxidant system.^265,266^ This phenomenon introduces novel anticancer drug discovery in the line of antioxidant therapy and treatment strategy. Therefore, anticancer drugs drive intracellular ROS production to destroy malignant cells. ROS levels increased by so-called oxidation therapy trigger cell death via the apoptosis or necrosis process.^267^ Several flavonoids such as quercetin,^268,269^ catechins,^270^ baicalein,^271^ delphinidin,^272^ apigenin, luteolin,^273^ and proanthocyanins protect the glial cells from oxidative stress, excitotoxicity, neuroinflammation, and cellular stress, although the increased level of free radicals reduces the proliferation of cells and even induces their death.^274–277^ These compounds also protect the brains of normal and cancer patients from ischemia. Gallic acid, an anticancer agent, can cause toxic effects by targeting mitochondrial antioxidant enzymes but also has beneficial effects on recovery of ischemic injuries.^278–280^ Overexpression of the oncogenic variant EGFRvIII and suppression of VEGF signaling are also involved in ROS production and represent an opportunity for the development of a new therapeutic strategy.^281,282^ Cardamonin (a chalcon) shows effective anti-inflammatory and anticarcinogenic activity in many cancers.^283,284^ It is reported that inhibition of NF-κB pathway activation is involved in breaking cellular redox homeostasis and triggers ROS production and accumulation through the JNK–mitogen-activated protein kinase (MAPK) axis.^285,286^ Due to high specificity and the power to cross the BBB, exosome- and nanovesicle-mediated delivery^287–289^ of peptides,^290–293^ small molecules, miRNA,^294–296^ and other drugs in both glioma and cerebral ischemia therapy has gained recent successes.

Hyperbaric oxygen (HBO) therapy is a recently developed procedure in which oxygen is used under an elevated atmospheric pressure, i.e., at a pressure higher than the pressure found on the surface of the earth at sea level, which is defined as 1 atm.^297^ Currently, hyperbaric oxygenation is extensively used as an adjunctive treatment for various diseases predominantly related to hypoxic and/or ischemic conditions. Because ischemic stroke and brain cancer are also related to hypoxia, HBO therapy has distinct effects on these diseases. Because the hypoxic regions in the tumor mass play a major role in tumor development and resistance to novel radio- and chemotherapies, HBO therapy offers a promising approach to overcoming oxygen insufficiency by increasing the oxygen supply to neoplastic tissue.^298–303^ Recent results clearly suggest that HBO does not induce cancer growth, recurrence, or metastasis. However, HBO is observed to have an inhibitory effect on neoplastic cell proliferation and to cause cancer cell apoptosis. The beneficial effect of HBO therapy varies with the tumor type, size of the lesion, and malignancy.^304–306^

Several drugs, e.g., sanguinarine,^307–309^ glycyrrhizin,^310^ piroxicam,^311–313^ salidroside,^314–316^ astragaloside,^317,318^ and others,^319–322^ are used in both glioma and ischemia treatment due to the counteracting effect of common signaling pathways.

**Fig. 4:**
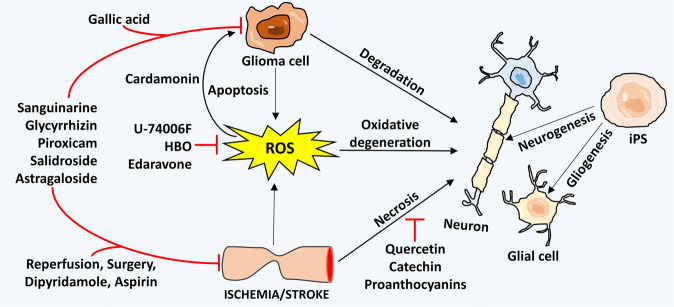
Selected common therapeutic approaches applied for both glioma and cerebral ischemia

Out of basic clinical need, several studies have been conducted to examine the remedial capability of either endogenous or transplanted stem cells in laboratory models of cerebral ischemic stroke. Further bolstering their good advantages, stem cells show the ability to react effectively to their condition, move to the zones of injury, and discharge neuroprotective compounds, notwithstanding their ability to create an assortment of new functional cell types.^323–325^ Such properties might manage their restorative and therapeutic potential in both the acute stage and also at a later time after ordinary medicinal treatments are no longer viable. Reconstruction after stroke via stem cells is not likely within a reasonable time frame, and extraordinary care must be taken to guarantee security before considering clinical trials. Preliminary pieces of evidence underpin the remedial capability of certain stem cells for treatment of ischemic damage in animal models^326–328^ (Fig. 4).

## Concluding remarks

The mechanisms underlying the development of stroke in glioma patients are not yet clearly defined. Patients who suffer from both cancer and stroke are more difficult to treat than stroke patients who do not have cancer. The survival rate of glioma patients is increasing with the development of anticancer medicines, nanotherapeutics, and improved targeted nanodelivery systems that easily cross the BBB. Treating stroke in glioma patients can be challenging, requires specific treatment strategies, and has clinical and pathological consequences. The characteristics, type, extent, and time interval from diagnosis of cancer and stroke might be important in the development of stroke in patients with glioma.
